# Soil microbial diversity and functional profiling of a tropical rainforest of a highly dissected low hill from the upper Itaya river basin revealed by analysis of shotgun metagenomics sequencing data

**DOI:** 10.1016/j.dib.2022.108205

**Published:** 2022-04-23

**Authors:** Marianela Cobos, Segundo L. Estela, Hicler N. Rodríguez, Carlos G. Castro, Miguel Grandez, Juan C. Castro

**Affiliations:** a Laboratorio de Biotecnología y Bioenergética (LBB), Universidad Científica del Perú (UCP), Iquitos, Perú; b Unidad Especializada del Laboratorio de Investigación en Biotecnología (UELIB), Centro de Investigaciones de Recursos Naturales de la UNAP (CIRNA), Universidad Nacional de la Amazonia Peruana (UNAP), Iquitos, Perú; c Departamento Académico de Ciencias Biomédicas y Biotecnología (DACBB), Facultad de Ciencias Biológicas (FCB), Universidad Nacional de la Amazonia Peruana (UNAP), Iquitos, Perú

**Keywords:** Peruvian amazon, Shotgun metagenomics, Soil microbiome, Tropical rainforest

## Abstract

The tropical rainforest of a highly dissected low hill from the upper Itaya river basin belongs to the western Amazonia region. Some investigations on the biodiversity of these rainforests were more focused on animals and plants diversity. The soils of this region are composed of moderately fertile sediments deposited recently from the initiation of the Andean orogenesis in the Miocene until now. However, scientific information about the soil microbial and functional diversity is still missing. This report presents shotgun metagenomics sequencing data from soils of this rainforest type. A composite loamy soil sample was collected from a primary forest, and metagenomic DNA was purified with standardized methods. Furthermore, libraries were prepared and paired-end sequenced on the Illumina NextSeq 550 platform. Raw Illumina paired-end reads have been uploaded and analysed in the Metagenomics RAST server (MG-RAST). The raw sequence data in fastq format is available at NCBI's Sequence Read Archive (SRA) with accession number SRX12846710.

## Specifications Table

**Table:** 

Subject	Microbiology: Microbiome
Specific subject area	Metagenomics
Type of data	Figures and shotgun metagenomics sequencing data
How data were acquired	Shotgun DNA sequencing using Illumina NextSeq 550 platform
Data format	Raw and Analysed
Description of data collection	A composite loamy soil sample (thirteen soil cores of ∼100 g) was collected from a primary forest of a highly dissected low hill from the upper Itaya river basin. Total microbial genomic DNA was purified from the composite soil sample using standardized methods, and shotgun metagenomic sequencing was performed using the Illumina NextSeq 550 platform (2 × 150 bp paired ends). Raw Illumina paired-end reads were uploaded and analysed in the Metagenomics RAST server (MG-RAST).
Data source location	Institutions: Universidad Nacional de la Amazonia Peruana and Universidad Científica del Perú City/Town/Region: Iquitos/Maynas/Loreto Region Country: Peru Latitude and longitude (and GPS coordinates) for collected samples/data: 4°15′46.69" S, 73°38′18.67" W
Data accessibility	Raw Illumina paired-end reads are available at NCBI under the study entitled “Genomic and Metagenomic Analysis of The Peruvian Amazon Microbial Diversity” (BioProject No. PRJNA769943, https://www.ncbi.nlm.nih.gov/bioproject/PRJNA769943) and SRA accession number: SRX12846710 (https://www.ncbi.nlm.nih.gov/sra/SRX12846710)

## Value of the Data

• This dataset provides valuable baseline information on the soil microbial diversity and functional profile of a tropical rainforest of a highly dissected low hill from the upper Itaya river basin of the Peruvian Amazon.
• This dataset could be a source of novel genes encoding proteins and enzymes useful for multiple biotechnological applications.
• This dataset can be used in comparative studies of different types of forests and associated soils. Also, it can be helpful to know the critical functions of the soil microbiome abundance and diversity in conserving soil health under climatic changes.

## 1 Data Description

**Fig. 1:**
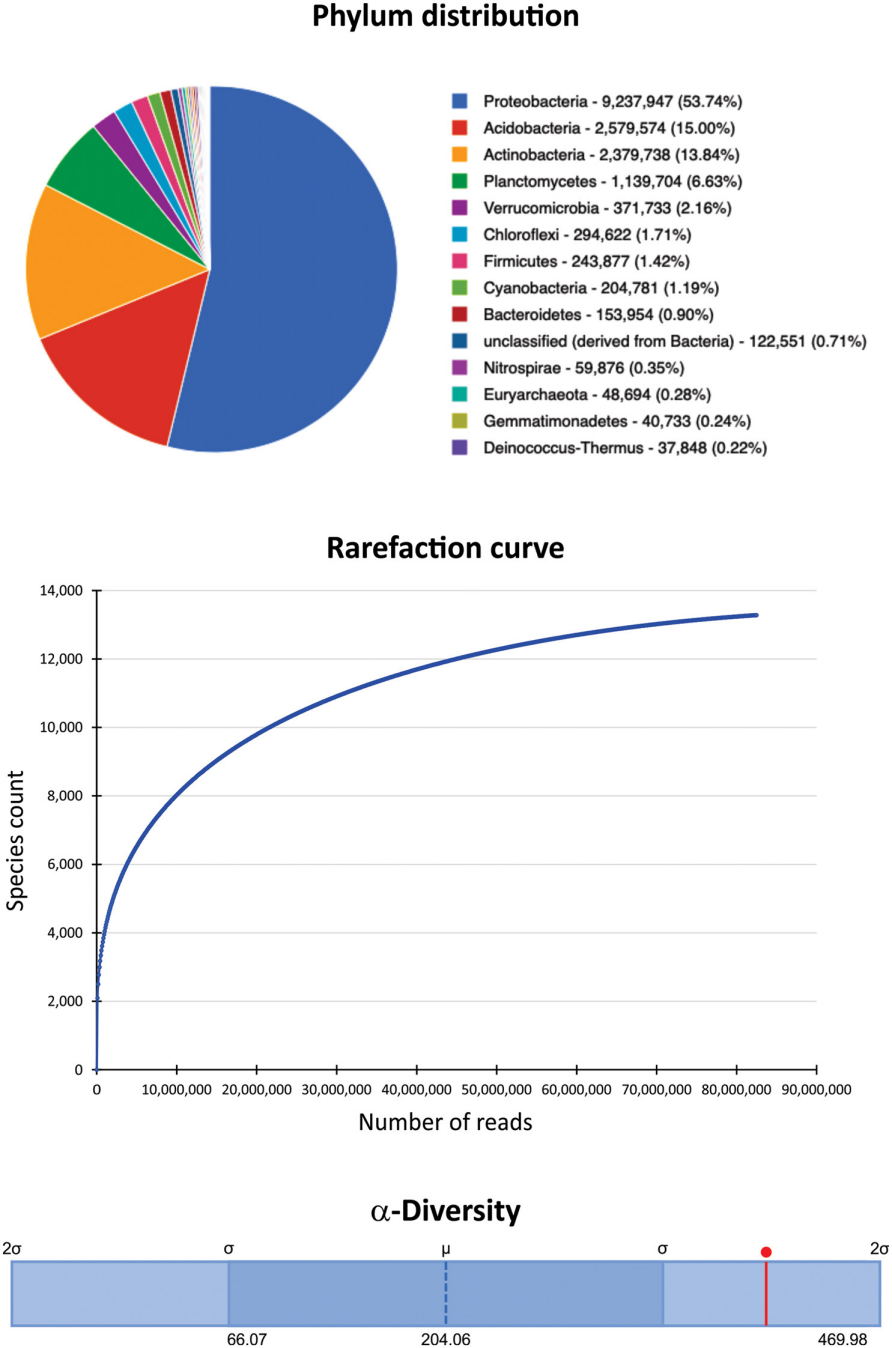
Phylum levels, rarefaction curve and α-diversity based on shotgun metagenomics of the microbiome from a composite soil sample collected from a primary forest of highly dissected low hill from the upper Itaya river basin.

**Fig. 2:**
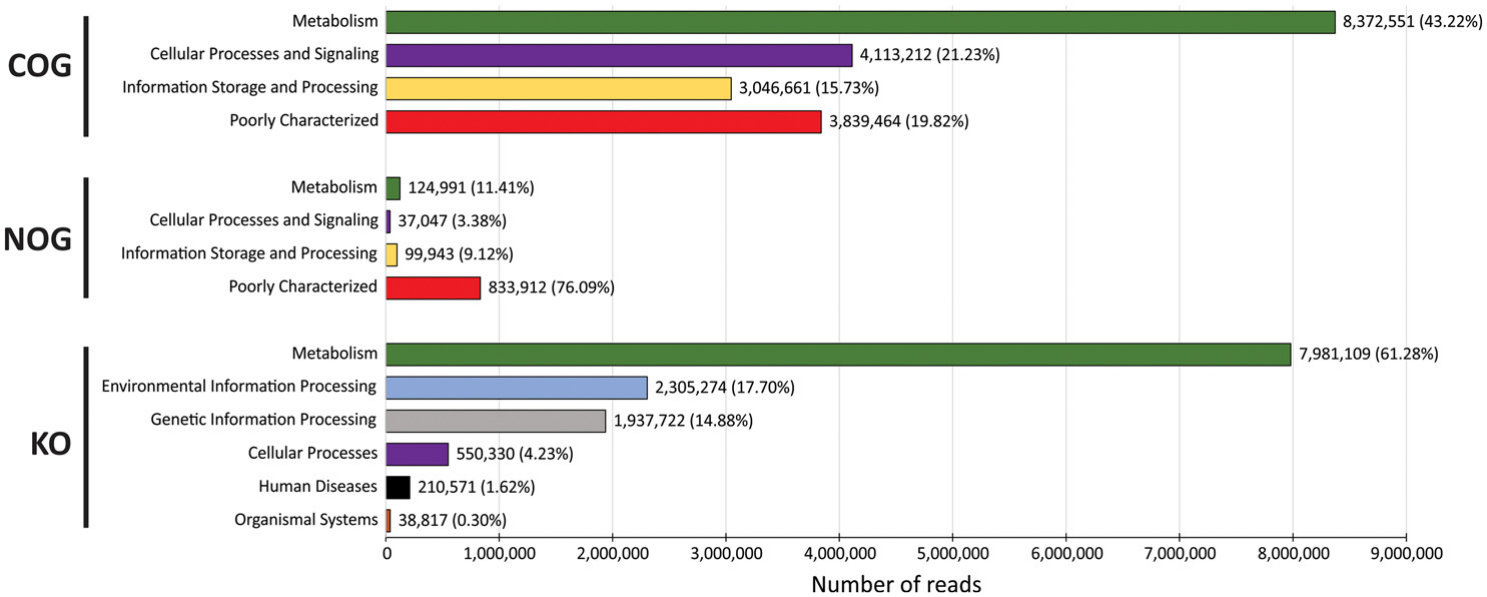
Potential functional categories for COGs, KOs, NOGs based on shotgun metagenomics of the microbiome from a composite soil sample collected from a primary forest of highly dissected low hill from the upper Itaya river basin.

**Fig. 3:**
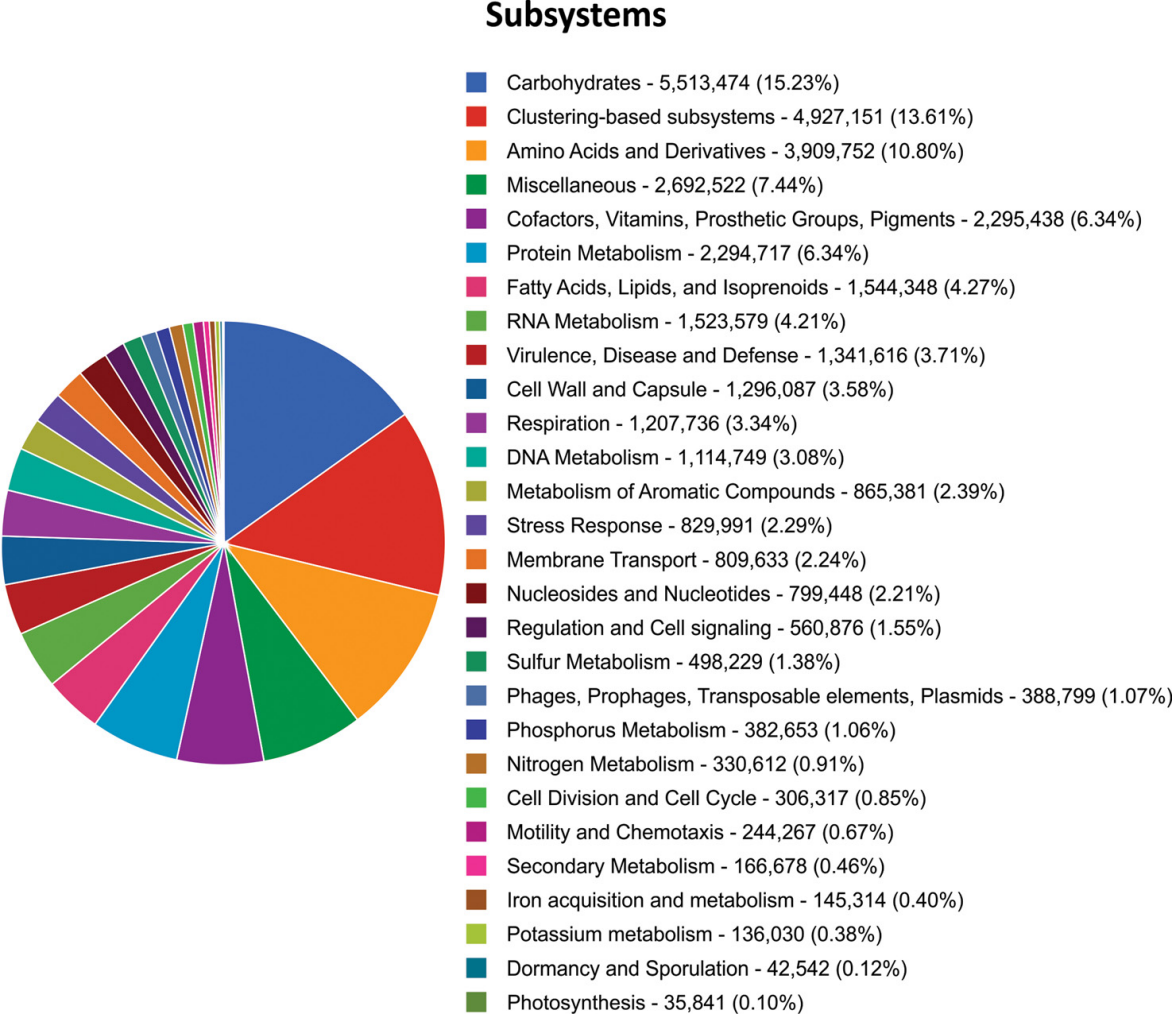
Potential functional categories at Subsystems level 1 based on shotgun metagenomics of the microbiome from a composite soil sample collected from a primary forest of highly dissected low hill from the upper Itaya river basin.

The dataset contains raw Illumina paired-end reads acquired through shotgun metagenomics sequencing of metagenomic DNA purified from a composite loamy soil sample collected from a primary forest of a highly dissected low hill from the upper Itaya river basin. The raw sequencing data contain 82,478,190 sequences totaling 12,3 Gbp with a mean sequence length of 150 ± 7 bp. The data files (reads in FASTQ format) were deposited at the NCBI database under the study entitled “Genomic and Metagenomic Analysis of The Peruvian Amazon Microbial Diversity”, BioProject No. PRJNA769943 (https://www.ncbi.nlm.nih.gov/bioproject/PRJNA769943), BioSample accession number: SAMN22794754 (https://www.ncbi.nlm.nih.gov/biosample/SAMN22794754) and SRA accession number: SRX12846710 (https://www.ncbi.nlm.nih.gov/sra/SRX12846710). MG-RAST analysis showed that 80,230,282 sequences passed the quality control; from these 4,732,368 sequences (5.90%) were unknown, and 75,497,914 sequences (94.10%) had predicted features. Of this last group, 66,812 sequences (0.09%) contain ribosomal RNA genes, 24,796,226 sequences (32.84%) contain predicted proteins with known functions, and 50,634,876 sequences (67.07%) contain predicted proteins with unknown function. About the taxonomic distribution, Bacteria (98.83%) and Archaea (0.55%) comprised most of the representative domains. The dataset includes data at phylum levels, rarefaction curve, and α-diversity (Fig. 1). Additionally, the dataset incorporates the distribution of potential functional categories for COGs, KOs, NOGs (Fig. 2), and Subsystems (Fig. 3) at the highest level supported by these functional hierarchies.

## 2 Experimental Design, Materials and Methods

### 2.1 Sample collection

For this dataset, a composite loamy soil sample was collected from a primary forest of a highly dissected low hill from the upper Itaya river basin (Supplementary Fig. S1), located in the Peruvian Amazon area of the Western Amazonia region, between 88 and 115 m.a.s.l. Loamy soil samples were obtained in the geographic coordinates 4°15′46.69" S and 73°38′18.67" W. Samples were obtained in March 2021 during the high-water level season. Thirteen soil cores of ∼100 g (10 cm in diameter and a depth range from 0 to 20 cm) were collected to capture a representative sample of soil microbial diversity at the site. The first soil core was designated the reference point for geographic coordinates. The twelve additional soil cores were sampled at five-meter intervals in each cardinal direction (N, S, W, and E), with three soil cores obtained in each direction. All thirteen core samples were pooled together, homogenized into a composite soil sample, and sieved through a 2 × 2 mm metal mesh sieve. The sieved soil was stored shortly at -80°C for further analysis.

### 2.2 Metagenomic DNA purification, library preparation, and shotgun sequencing

Metagenomic DNA was purified from the composite soil sample using the DNeasy® PowerSoil Pro Kit (Qiagen, Germany), following the manufacturer's instructions. The quality and quantity of the purified metagenomic DNA were determined using a NanoDrop 2000 spectrophotometer (Thermo Fisher Scientific, USA). Also, the purified metagenomic DNA concentration was assayed with the Qubit™ dsDNA BR Assay Kit using a Qubit™ 4 Fluorometer (Thermo Fisher Scientific, USA).

Libraries were prepared using the Nextera XT DNA Library Preparation Kit (Illumina, USA), following the manufacturer's instructions. First, metagenomic DNA was fragmented and tagged using a tagmentation process. Second, tagmented DNA was amplified using a limited-cycle PCR program to add the index adapters. Further, libraries were cleaned up using a 0.8x Agencourt® AMPure XP bead purification (Beckman Coulter, USA), and its sizes were verified with an Agilent High Sensitivity DNA Kit using an Agilent 2100 Bioanalyzer microfluidic electrophoresis (Agilent Technologies, USA). Finally, libraries were quantified using the Qubit™ dsDNA HS Assay Kit (Thermo Fisher Scientific) and paired-end (2 × 150 bp) sequenced with the Illumina NexSeq 550 platform.

### 2.3 Sequence analysis

Raw Illumina paired-end reads were uploaded as FASTQ files and analysed using the MG-RAST server v 4.0.3 [1], [2], [3] High-quality reads were subjected to analysis to predict, identify, and assign biological functions (gene annotations) to proteins and rRNA and then designate the functional categories using the COG ontology [4], NOG ontology, KEGG orthologs ontology, and Subsystems ontology. Taxonomic analysis was accomplished using data from the M5NR database [5]. Finally, the MG-RAST pipeline produced the sequence coverage by rarefaction analysis and the alpha diversity of species.

## CRediT authorship contribution statement

**Marianela Cobos:** Conceptualization, Supervision, Project administration, Resources, Funding acquisition, Writing – original draft. **Segundo L. Estela:** Methodology, Investigation, Formal analysis. **Hicler N. Rodríguez:** Methodology, Investigation, Data curation. **Carlos G. Castro:** Methodology, Software, Visualization. **Miguel Grandez:** Methodology, Investigation, Formal analysis. **Juan C. Castro:** Conceptualization, Funding acquisition, Software, Data curation, Writing – review & editing.

## Declaration of Competing Interest

The authors declare that they have no known competing financial interests or personal relationships which have, or could be perceived to have, influenced the work reported in this article.

## Data Availability

Metagenome from soil of the ACCARI-UCP (Original data) (Sequence Read Archive (SRA) - National Center For Biotechnology Information).
